# Instability of Plastid DNA in the Nuclear Genome

**DOI:** 10.1371/journal.pgen.1000323

**Published:** 2009-01-02

**Authors:** Anna E. Sheppard, Jeremy N. Timmis

**Affiliations:** School of Molecular and Biomedical Science, The University of Adelaide, South Australia, Australia; The University of North Carolina at Chapel Hill, United States of America

## Abstract

Functional gene transfer from the plastid (chloroplast) and mitochondrial genomes to the nucleus has been an important driving force in eukaryotic evolution. Non-functional DNA transfer is far more frequent, and the frequency of such transfers from the plastid to the nucleus has been determined experimentally in tobacco using transplastomic lines containing, in their plastid genome, a kanamycin resistance gene (*neo*) readymade for nuclear expression. Contrary to expectations, non-Mendelian segregation of the kanamycin resistance phenotype is seen in progeny of some lines in which *neo* has been transferred to the nuclear genome. Here, we provide a detailed analysis of the instability of kanamycin resistance in nine of these lines, and we show that it is due to deletion of *neo*. Four lines showed instability with variation between progeny derived from different areas of the same plant, suggesting a loss of *neo* during somatic cell division. One line showed a consistent reduction in the proportion of kanamycin-resistant progeny, suggesting a loss of *neo* during meiosis, and the remaining four lines were relatively stable. To avoid genomic enlargement, the high frequency of plastid DNA integration into the nuclear genome necessitates a counterbalancing removal process. This is the first demonstration of such loss involving a high proportion of recent nuclear integrants. We propose that insertion, deletion, and rearrangement of plastid sequences in the nuclear genome are important evolutionary processes in the generation of novel nuclear genes. This work is also relevant in the context of transgenic plant research and crop production, because similar processes to those described here may be involved in the loss of plant transgenes.

## Author Summary

In eukaryotes, mitochondria and plastids are the descendents of once free-living prokaryotic ancestors. Over time, these organelles have donated a great deal of genetic material to the nuclear genome. Although usually non-functional, these DNA transfer events have, over evolutionary time, resulted in a large pool of functional nuclear genes and therefore the process of DNA transfer has been an important driving force in eukaryotic evolution. Previous studies showed that DNA transfer of a specific marker gene (*neo*) from the plastid to the nucleus occurred in one in every 11,000 to 16,000 male gametes. Because of this high frequency of transfer and the large size of integrants, this process would be expected to result in a cumulative increase in genome size, unless there are counterbalancing deletion events. In this study, we analysed the stability of the *neo* gene after integration into the nuclear genome. We found that the gene is highly unstable, with deletion often occurring within a single generation. These results indicate that plastid DNA insertion into and removal from the nuclear genome are in dynamic equilibrium, thus providing a mechanism by which the chances of functional DNA insertion are maximised without compromising the nuclear genome as a whole.

## Introduction

In eukaryotes, plastids and mitochondria are derived from once free living cyanobacteria and α-proteobacteria respectively [1],[2]. Over evolutionary time, many of their genes have been relocated to the nuclear genome and in many cases this is an ongoing process [3]–[5]. Such functional gene transfer is not a trivial process and is dependent on several steps. The DNA sequence encoding the gene must not only integrate into the nuclear genome, but also it must acquire appropriate regulatory sequences for expression in the nucleus. Although an organellar sequence may occasionally integrate directly into a fortuitous location in the nuclear genome and become immediately functional, it is likely that most functional gene transfer events involve postinsertional rearrangements that bring the organellar gene into the context of a nuclear promoter [6]. In many cases these transfers involve gene products that retain their original function and are targeted back to the appropriate organelle and such genes must also acquire a transit peptide-encoding sequence. However, the original organellar function is not always maintained. For example, in Arabidopsis it has been estimated that approximately 18% (4,500) of nuclear genes are plastid-derived, and a large proportion of their products are not targeted to the plastid [7]. In algae this is also the case, although a lower proportion of ancestral cyanobacterial genes appear to have assumed non-plastid functions [8]. Therefore, organellar genomes have been a significant source of new genes in eukaryotic evolution.

While functional gene transfers from the plastid to the nuclear genome are relatively rare, non-functional sequence transfer occurs much more frequently and many nuclear genomes are riddled with such sequences, designated *nupts* (nuclear integrants of plastid DNA) [9]. The frequency of *nupt* formation has been measured experimentally in *Nicotiana tabacum* using transplastomic lines containing in their plastid genome a kanamycin resistance gene (*neo*) under the control of nuclear regulatory sequences, so that kanamycin selection can be used to detect transfer of *neo* to the nuclear genome. From these experiments it has been estimated that the frequency of transfer in the male germline is approximately 1 event per 11,000 to 16,000 pollen grains [10],[11], while the frequencies of transfer in the female germline and in somatic cells appear to be much lower [11],[12]. A number of the kanamycin resistant (kr) lines derived from the former experiments have been partially characterised at the molecular level and their causative experimental *nupts* are characteristically tens of kilobases in size [13]. The high frequency of plastid DNA (ptDNA) integration into the nuclear genome, together with the typically large size of the integrants, suggests the occurrence of counterbalancing removal events that would prevent a progressive increase in nuclear genome size. In fact, genome-wide analyses have revealed that decay of plastid sequences in the nuclear genome occurs relatively quickly in evolutionary terms [9]. With the experimental kr lines now available we have new tools with which to analyse any loss or decay that may occur within one or a few generations. Some of these kr lines were previously found to be unstable with respect to the kanamycin resistance phenotype in that there was a deficiency of kanamycin resistant progeny compared with Mendelian expectations [10]. Here we provide a detailed analysis of this instability in nine new kr lines [11] and we show that it is due to deletion of *neo*.

## Results

### Phenotypic Analysis of Instability

To investigate the genetic stability of kanamycin resistance seen in newly transposed *nupts* containing the *neo* gene, we analysed nine kr plants (kr2.1–2.7, kr2.9 and kr2.10), each of which resulted from an independent transposition of ptDNA to the nucleus [11]. Preliminary work indicated that different seed capsules from the same plant sometimes gave rise to different ratios of kanamycin resistant to kanamycin sensitive (kr∶ks) progeny. This suggested that the results presented by Huang *et al.* [10] provided an incomplete picture of the nature of instability. Therefore we determined the proportion of kanamycin resistant progeny arising from a large number of individual self-fertilised seed capsules for each of the nine independent kr plants.

**Figure 1:**
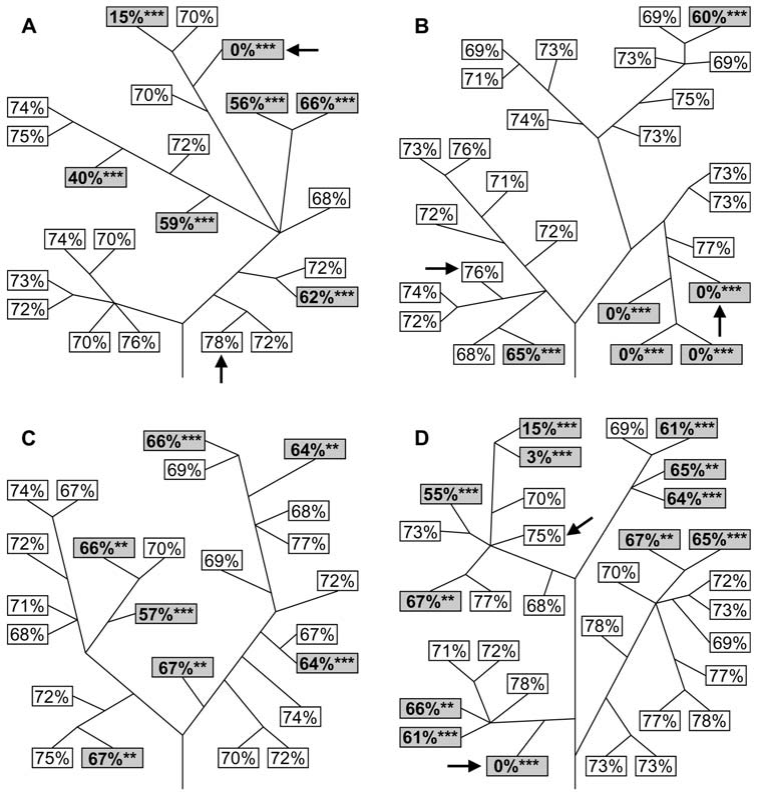
Instability of kanamycin resistance. The percentages of kanamycin resistant progeny from a number of self-fertilised seed capsules are shown for kr2.3 (A), kr2.5 (B), kr2.7 (C) and kr2.10 (D). Each box represents a seed capsule, with the percentage of kanamycin resistant progeny from that capsule shown. Approximately 200–250 seeds from each capsule were tested for kanamycin resistance. Lines represent branches (not to scale) and are included to show the branching pattern of the plants from which individual seed capsules were progeny tested. Seed capsules that deviate significantly (*P*<0.01) from the expected 75% kanamycin resistant progeny are highlighted with grey shading. Arrows indicate seed capsules which were used for PCR and DNA blot analysis (see Figures 3 and 4). ** *P*<0.01, *** *P*<0.001.

For four of these plants (kr2.1, kr2.2, kr2.4 and kr2.6), all seed capsules gave the expected 3∶1 Mendelian ratio of kr∶ks progeny (Table 1), indicating that kanamycin resistance in these lines was relatively stable. Four plants (kr2.3, kr2.5, kr2.7 and kr2.10) showed variability between seed capsules of the same plant (Table 1 and Figure 1), with seed from some capsules showing the expected 3∶1 ratio and others showing a significant reduction in the proportion of resistant progeny. The severity of this reduction was variable between seed capsules, ranging from the statistical threshold of detection to a complete loss of kanamycin resistance. There appeared to be a loose clustering of seed capsules with aberrant ratios (Figure 1), suggesting that, at least in some cases, kanamycin resistance was lost somatically in the cell lineages leading to those seed capsules. The ninth plant, kr2.9, showed a significant reduction in the proportion of kanamycin resistant progeny in all seed capsules tested, with ratios approximating 1∶1 instead of the expected 3∶1 kr∶ks (Table 1).

**Table 1:** Segregation of kanamycin resistance in individual self-fertilised seed capsules of kr2.1-2.10.

	Seed Capsules	Highest %R (*P*)	Lowest %R (*P*)
kr2.1	31	78% (NS)	69% (NS)
kr2.2	26	80% (NS)	70% (**)^a^
kr2.3	22	78% (NS)	0% (***)
kr2.4	25	82% (NS)	68% (NS)
kr2.5	27	77% (NS)	0% (***)
kr2.6	18	78% (NS)	71% (NS)
kr2.7	24	77% (NS)	57% (***)
kr2.9	39	60% (***)	44% (***)
kr2.10	31	78% (NS)	0% (***)

The percentage of kanamycin resistant progeny from the seed capsules which gave the highest and lowest kr∶ks ratios for each of kr2.1-2.10 are shown. Approximately 200–250 seeds from each capsule were tested for kanamycin resistance. *P* values correspond to deviation from 3∶1 kr∶ks.

NS *P*>0.01, ** *P*<0.01, *** *P*<0.001.

a This is the only seed capsule of kr2.2 which deviated significantly from 75% kanamycin resistance, so given the level of significance it is likely to represent random variation rather than a biological effect.

When the seed capsules that deviated significantly from 3∶1 kr∶ks were excluded and the data for the remaining seed capsules of each plant combined to give larger numbers for analysis, the progeny of three of the four unstable plants (kr2.3, kr2.5 and kr2.7) showed a statistically significant deviation from 3∶1 kr∶ks (Table 2). In all cases this was caused by a reduction in the proportion of kanamycin resistant progeny, indicating that instability was also playing a role in some of the seed capsules which initially appeared to be segregating normally. A paucity of resistant progeny was also revealed for the pooled data of kr2.10, though the reduction was not significant. This is surprising as the proportion of capsules showing significant deviation from 3∶1 kr∶ks was highest in this line. Although analyses of individual capsules of kr2.1 suggested normal Mendelian inheritance, when the data from all its seed capsules were combined, a significant deviation from 3∶1 kr∶ks was revealed, indicating that this line shows some instability. In contrast, no significant instability could be detected by pooling data for kr2.2, kr2.4 or kr2.6.

**Table 2:** Overall segregation of kanamycin resistance in self-fertilised progeny of kr2.1-2.10.

	Resistant	Sensitive	*P*
kr2.1	6479	2327	**
kr2.2^a^	5342	1840	NS
kr2.3	2974	1127	***
kr2.4	3925	1366	NS
kr2.5	3770	1429	***
kr2.6	3336	1149	NS
kr2.7	2601	1050	***
kr2.10	3090	1124	NS

The total number of kanamycin resistant and sensitive seedlings from all seed capsules of kr2.1-2.10, excluding those which deviated significantly (*P*<0.01) from 3∶1 kr∶ks, are shown. *P* values correspond to deviation from 3∶1 kr∶ks.

NS *P*>0.01, ** *P*<0.01, *** *P*<0.001.

a In this case the one seed capsule which deviated significantly from 3∶1 kr∶ks was included in the analysis (see Table 1).

**Figure 2:**
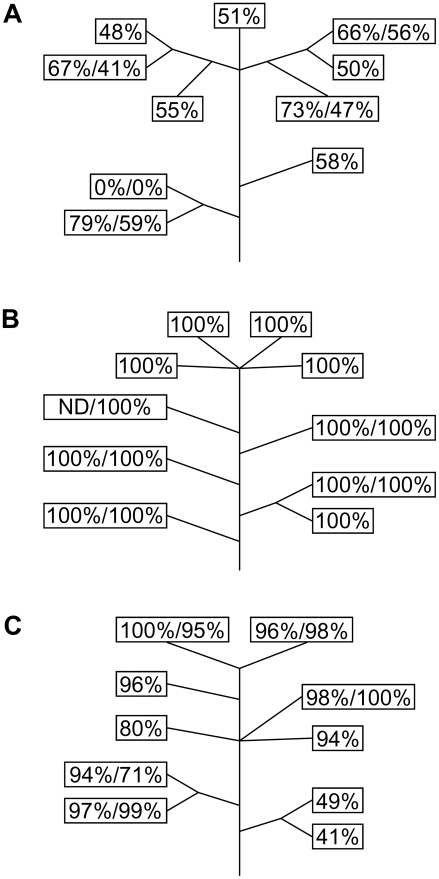
Analysis of instability in homozygous descendents of kr2.3 (A), kr2.7 (B) and kr2.10 (C). Each box represents a seed capsule. Where one number is shown, it indicates the percentage of kanamycin resistant progeny from a backcross to male wildtype. Where two numbers are shown, that flower was used for both self-fertilisation and backcrossing to female wildtype. The numbers indicate the percentage of kanamycin resistant progeny from self-fertilisation and backcrossing respectively. Each number represents approximately 100–150 seeds which were tested for kanamycin resistance. Lines represent branches (not to scale) and are included to show the branching pattern of the plants from which individual seed capsules were progeny tested. ND not determined.

Homozygous lines of kr2.3, kr2.7 and kr2.10 were obtained by growing self-fertilised progeny of the original hemizygous plants and selecting those which gave 100% kanamycin resistant progeny after self-fertilisation. Individual plants from these 100% resistant progeny (one for each kr line) were then grown to maturity and self-fertilised and backcrossed progeny were tested for kanamycin resistance. The homozygous kr2.3 plant behaved in a similar way to the original hemizygous plant; some crosses segregated according to Mendelian expectations for a hemizygote, while others showed a reduced proportion of kanamycin resistant progeny (Figure 2A). None showed a significant increase in the proportion of kanamycin resistant progeny compared with expectations for a hemizygote. Therefore, it is most likely that one copy of *neo* was lost early in the development of this plant, causing an initially homozygous plant to become hemizygous. All progeny from the homozygous kr2.7 plant were resistant to kanamycin, so instability could not be detected in this case (Figure 2B). The homozygous kr2.10 plant gave some kanamycin sensitive progeny from most crosses, but kanamycin resistant percentages were close to 100% (Figure 2C). Therefore, in this case we can be very confident that the instability of kanamycin resistance was occurring in a homozygote. All these data indicate that the instability is maintained in descendents of the original kr plants and that it is not affected by homozygosity. Furthermore, the instability does not appear to be affected by the type of cross (self-fertilisation or backcrossing) or by the direction of backcrossing (Figure 2). Consistent with results for the initial kr plants (Figure 1), the kr2.7 line appears to be more stable than kr2.3 or kr2.10.

### The Molecular Basis of Instability

**Figure 3:**
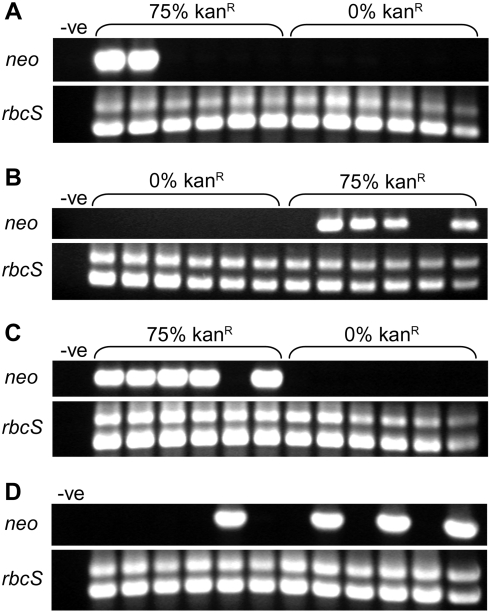
PCR analysis of kr lines with reduced levels of kanamycin resistance. A–C. PCR of *neo* from 6 plants grown from seeds of a normally segregating self-fertilised seed capsule (75% kan^R^) and 6 plants grown from seeds of a capsule which showed no kanamycin resistant progeny (0% kan^R^) for each of kr2.3 (A), kr2.5 (B) and kr2.10 (C) (see Figure 1). D. PCR of *neo* from progeny of a self-fertilised seed capsule of kr2.9 which gave 50% kanamycin resistant progeny. Results for 12 plants are shown; this is representative of a larger experiment in which 36 plants were tested (see text). In all cases control PCRs with *rbcS* primers are also shown. The *neo* primers amplify a single product of approximately 800 bp. The *rbcS* primers amplify two products of approximately 850 bp and 1 kb. -ve no template.

**Figure 4:**
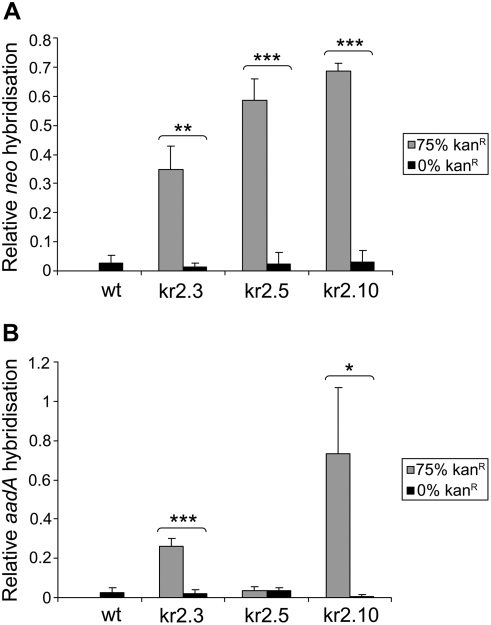
Quantification of DNA blot analysis. DNA slot blotting was performed using pooled DNA from at least 20 plants grown from seeds of a normally segregating self-fertilised seed capsule (75% kan^R^) and pooled DNA from at least 20 plants grown from a capsule which showed no kanamycin resistant progeny (0% kan^R^) for each of kr2.3, kr2.5 and kr2.10 (see Figure 1), as well as a wildtype control. Triplicates of each sample were probed with *neo* or *aadA* probes, followed by probing with ribosomal DNA as a loading control. The graphs show average hybridisation to *neo* (A) or *aadA* (B) after normalisation to ribosomal DNA hybridisation. Error bars show standard deviation. * *P*<0.05 ** *P*<0.01, *** *P*<0.001; Student's *t*-test.

**Figure 5:**
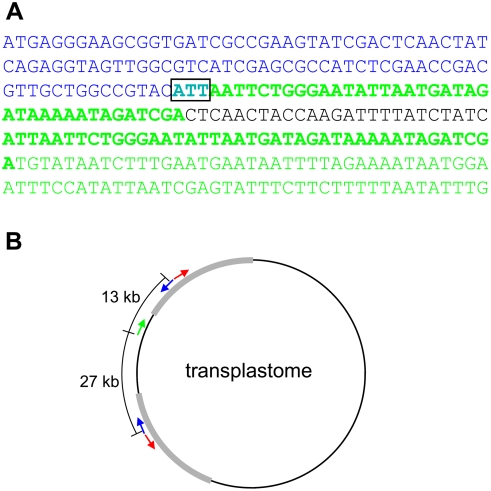
Origin and partial sequence of the kr2.5 nuclear integrant. A. Partial sequence of the kr2.5 nuclear integrant. Blue text indicates *aadA* sequence, beginning from the start codon, and green text indicates plastid sequence, with the two bold regions being identical. The box highlights a 3 bp region of overlap between *aadA* and plastid sequences. Black text indicates non-plastid sequence of unknown origin. B. Structure of the transplastome showing the origin of kr2.5 nuclear integrant sequences (not to scale). *Neo* and *aadA* are represented by red and blue arrows respectively. They are present in two copies as they are located within the inverted repeats (shown in grey). The green arrow indicates plastid sequence adjacent to *aadA* in the kr2.5 nuclear integrant. In each case, the direction of the arrow corresponds to the direction of the sequence as written in part A.

Loss of kanamycin resistance could be caused by a genetic change in the *neo* gene (deletion or sequence decay), or by silencing of the gene through epigenetic mechanisms, a phenomenon which is commonly observed with plant transgenes [14]. To distinguish between these possibilities, progeny from seed capsules of kr2.3, kr2.5 and kr2.10 which had completely lost kanamycin resistance (Figure 1) were analysed for the presence of *neo* by PCR (Figure 3A–C). In all three cases, *neo* could not be amplified using primers designed to amplify most of the gene, suggesting that at least part of it had been deleted. In contrast the primers were able to amplify the target sequences from sibling plants derived from normally segregating seed capsules. Further analyses with alternative primers designed to amplify smaller fragments also suggested that extensive deletion was involved (data not shown). Therefore, hybridisation experiments were performed, which confirmed that the *neo* gene had been lost (Figure 4A). In addition, probing was performed with *aadA*, a gene which was initially used for selection of transplastomic lines [10] and which was co-transferred in whole or part to the nucleus in kr2.3, kr2.5 and kr2.10 [11]. In progeny of kr2.3 and kr2.10 *aadA* was also lost, suggesting deletions of at least 2.4 kb encompassing both genes (Figure 4B). In the kr2.5 integrant, only a small fragment of *aadA* is present (Figure 5; see below) and consequently it was not detected by the hybridisation experiments (Figure 4B). Therefore it could not be determined whether the partial gene was lost in this case.

Progeny of kr2.9, the plant that consistently gave ∼50% resistant progeny from self-fertilisation, were also analysed for the presence of *neo* by PCR. Since there were no seed capsules where kanamycin resistance had been completely lost, 36 seeds from a typical, previously tested, capsule were grown in the absence of selection and assayed individually by PCR (Figure 3D). Thirteen of these gave a positive result, which deviates significantly (*P*<0.0001) from the 75% that would be expected if epigenetic silencing were the mechanism responsible. Therefore, the instability in kr2.9 is also due to the loss of *neo*, even though it behaves in quite a different way to the other unstable kr plants (Table 1).

### Sequence Analysis of Integration Sites

Based on the above results, the loss of *neo* must occur by deletion of some or all of the *nupt* or by such a high level of sequence decay that specific probes and primers are no longer able to recognise the remaining sequences. Clearly, it would be useful to know what sequences are involved in plastid sequence integration in the nuclear genome and subsequent deletion and / or decay. Ideally we would like to know the sequence of the pre-insertion site (i.e. the sequence present prior to insertion), the sequence of the whole integrant and what remains following deletion. However, determining these sequences is not a trivial process. Even the first step (of determining the pre-insertion site) is a challenging task. The reason for this is that the integrants are often very large, with *neo* and *aadA* being flanked by many kilobases of cointegrated ptDNA [10],[11],[13]. Techniques that are generally used for determining flanking sequence in transgenic lines, such as genome walking and thermal asymmetric interlaced (TAIL-) PCR, have two limitations that prevent their use in this context (except in special cases, see below). They require the use of a primer that binds uniquely to the target sequence (i.e. the primer binding site must not be present elsewhere in the genome) and the amount of sequence information that can be obtained is generally only up to a few kilobases from the primer binding site. Since ptDNA sequences are present in high copy number in the nuclear genome as pre-existing *nupts* [9],[15], primers that bind within ptDNA are useless for genome walking and TAIL-PCR. Therefore, these approaches can only utilise primers that bind within *neo* or *aadA*. As the amount of ptDNA flanking these marker genes exceeds the amount of sequence information that can be obtained using these techniques in the vast majority of cases, these approaches are not useful for determining junction sequences beyond the ptDNA immediately flanking *neo* and *aadA*. Therefore, they cannot be used in determining the pre-insertion site unless one of the marker genes is very close to the integrant boundary.

In kr2.5, PCR results indicated that *aadA* was truncated (data not shown), so in this special case it was possible to determine the adjacent sequence using TAIL-PCR. This sequence is composed of 41 bp of ptDNA, 25 bp of unidentified sequence, presumed to be derived from the nuclear genome, and >1.1 kb of continuous ptDNA with the first 41 bp being identical to that described above (Figure 5A). Standard PCR using a more distant ptDNA primer indicated that this ptDNA sequence continues for at least 2.6 kb. Interestingly, the ptDNA sequence is from a region at least 13 kb away from *aadA* in the transplastome (Figure 5B). Another feature of this sequence is a 3 bp region of microhomology at the *aadA* / ptDNA junction (Figure 5A). Microhomology is a characteristic of illegitimate recombination and is often found at *nupt* junctions [13]. Because the sequence adjacent to *aadA* is ptDNA, albeit distant from *aadA* in the transplastome, TAIL-PCR cannot be used to obtain any further information about the sequence of the kr2.5 integrant or its pre-insertion site, for the same reasons as described above.

An alternative method that can yield sequence information more distant from the reporter genes is inverse PCR. However, this method also has its limitations as at least one primer binding site must be within *neo* or *aadA*. Therefore obtaining sequence data for larger integrants can pose serious technical difficulties. Another barrier to determining pre-insertion sites is that the integrants are often quite complex [13], with various rearrangements that have occurred during, or subsequent to, integration. Nevertheless, inverse PCR has been used with some success to determine border sequences for previously generated kr lines, although this has mainly been limited to smaller integrants and internal border sequences [10],[13]. Despite this partial success, it has not been possible to confirm a single pre-insertion site, let alone the complete integrant sequence or its remnants after deletion in the case of an unstable kr line. Also, the sequence information that has been determined previously is essentially limited to kr lines which appear to be stable. Furthermore, the kr lines for which *neo* deletion has been confirmed (kr2.3, kr2.5 and kr2.10) appear to have particularly large integrants on the basis of Southern analysis [11]. This makes them even more difficult to work with than the kr lines for which partial success has been reported previously [10],[13]. For these reasons we have not been able to further describe the process of instability at the sequence level.

## Discussion

We have shown that instability of kanamycin resistance in kr2.3, kr2.5 and kr2.10 is due to the absence of *neo*. Chimerism of the initial kr plants as an explanation for this is ruled out by the instability also being present in subsequent generations. Somatic recombination is ruled out by the observation that homozygous plants are also unstable. Therefore, the instability must be caused by the loss of *neo* due to deletion of some or all of the chromosomal sequence containing the integrant or its large-scale degeneration. It is disappointing that we have not been able to obtain sequence information to shed light on the mechanism of loss. Analyses of organelle DNA insertions in the nuclear genomes of Arabidopsis and rice have suggested that deletions occur by replication slippage, as deleted fragments are often flanked by short direct repeats [16],[17]. However, the largest deletions observed in these studies were only a few hundred base pairs long. Therefore, it is not clear whether the same mechanisms are involved here, as the complete loss of *neo* and *aadA* would require a much larger deletion. We have invested much time in investigating the process of insertion and deletion at the sequence level, with minimal success. It appears that the most fruitful approach would be to construct individual BAC libraries for each unstable and deleted genotype, but even this approach could be problematic because the DNA used for construction of the libraries would be subject to variation as a result of the instability.

The under representation of kanamycin resistant progeny from kr2.9 also is due to the absence of *neo*, but it may be caused by a different underlying mechanism, since the kr∶ks ratio is consistently altered to approximately 1∶1 in self-fertilised progeny. One possibility for this could be non-transmission of *neo* through either the male or the female germline. However, backcrossing kr2.9 to male wildtype gave 24% resistant progeny (n = 232), which is not consistent with this explanation. Since the kr∶ks ratio was found to be approximately the same in 39 self-fertilisations, it seems likely that *neo* is mitotically stable but meiotically unstable, with approximately 50% loss during both male and female meiosis. This loss could be occurring by a gene conversion-like process where a template is used to ‘correct’ the kr2.9 integrant sequence, resulting in the removal of *neo*. The template could be the native homologous or homeologous allele (*N. tabacum* is an allotetraploid), or an adjacent pre-existing *nupt* with homology to the native ptDNA that flanks *neo* in the experimental construct.

The kr2.5 integrant has been partially characterised and *aadA* is adjacent to a *nupt* sequence which is physically distant from *aadA* in the transplastome. There are several possible explanations for this observation. Firstly, this plastid sequence may be part of a pre-existing *nupt*. However, sequencing of 2.6 kb revealed perfect identity to the plastid genome (data not shown), indicating that this sequence is of very recent origin in the nucleus. Secondly, it may have integrated into the nuclear genome as part of the same event as *neo* and *aadA* with rearrangement occurring at the time of integration. Finally, it may have integrated into the nuclear genome with *neo* and *aadA* as a continuous sequence from the transplastome and subsequent rearrangement and / or deletion may have then brought it into the vicinity of *aadA*. This final possibility could be explained by mechanisms similar to those envisaged for the deletion of *neo*.

Why do some kr lines show a high level of instability, while others appear to be more stable? One possibility is that the chromosomal location and sequence context of the integrant determines the level of stability. For example, nuclear integration of organellar sequences may be dependent on the formation of double strand breaks (DSBs) [13],[18],[19] and if some regions of the genome are particularly prone to DSBs, as is the case for meiotic recombination hotspots in yeast [20], this could facilitate both integration and removal of *nupts* in these regions. Differing levels of stability could represent differing tendencies to sustain DSBs. Another possibility is that the level of stability depends on the sequence of the integrant itself, rather than the surrounding sequence. In this case *nupts* may be recognised as foreign DNA and subsequently removed. For example, the recognition could occur via differences in methylation status, as plant nuclear DNA is highly methylated and ptDNA is not [21],[22]. Certain plastid sequences may be more prone to elimination than others, or alternatively the level of stability may depend on the size of the integrant. Differing levels of stability also may be related to differences in transgene copy number. Kr2.3, kr2.5, kr2.7 and kr2.10 all display instability, but kr2.7 appears to be the least unstable of this group (Figures 1 and 2). Southern blotting indicates that kr2.3, kr2.5 and kr2.10 have single or low copy insertions while kr2.7 appears to have several copies of *neo* [11]. Therefore it may be that in the case of kr2.7, the loss of kanamycin resistance requires several deletion events or an infrequent large deletion.

Some nuclear genomes contain large numbers of *nupts* [9]. The high level of deletion that has been observed in this study raises the question of why supposedly non-functional *nupts* are retained in nuclear genomes for long periods. Firstly, it appears that some integrants are more stable than others in terms of deletion frequency, so it is possible that a small proportion of *nupts* are retained by chance because deletion occurs only very rarely. In support of this idea, bioinformatic analysis has indicated that recent *nupt* insertions are far more prevalent than older ones as assessed by their close similarity to extant *bona fide* ptDNA [9]. It is also possible that selection plays a role in the retention of some *nupts*. Clearly if a *nupt* is functional and it confers a selective advantage then it is likely to be retained even in the presence of some level of genomic instability. Therefore, some *nupts* that have been retained in nuclear genomes may have as yet unidentified functional significance. In addition, a *nupt* that integrates near a gene or other functionally significant region of the genome may be retained even though the *nupt* itself does not confer a selective advantage because deletions would tend to disrupt the nearby functional sequences and therefore be selected against.

Physical loss of transgenes has been reported in a wide range of plants [23]–[28], but little is known about the causes and mechanisms involved in transgene elimination. Furthermore, we are not aware of any studies where variation in transgene instability has been examined within a single plant. Therefore it is possible that similar mechanisms are involved in the removal of both transgenes and *nupts*. In this case, it may be that transgenic lines which appear to be stable on the basis of limited progeny testing are actually relatively unstable (as in Figure 1), an undesirable trait in biotechnological applications. Therefore the work described here is relevant not only in the context of endosymbiotic evolution, but also in the broader context of transgenic plant research and crop production.

Previous studies have shown that ptDNA is integrated into the nucleus at high frequencies that can be measured in the laboratory [10]–[12]. Here we have shown that, in some cases, newly integrated ptDNA is also removed from the nuclear genome at high frequency within a single generation. Presumably a more thorough analysis over many generations would reveal losses in other lines as well. This provides an explanation for the avoidance of increasing genome size in the presence of such a high transfer frequency. What is the functional significance of having such high frequencies of ptDNA integration and subsequent deletion? Many nuclear genes are plastid derived [7] so it is feasible that a high transfer frequency provides more opportunity for the evolution of these plastid-derived nuclear genes. However, it is clear that the vast majority of ptDNA integrations result in non-functional sequences. Therefore, if deletions and rearrangements involving part of the integrant and / or flanking sequence are frequent, this not only counterbalances the problem of increasing genome size associated with a large transfer frequency, but also provides many more opportunities for ptDNA to attain functional sequence contexts in the nucleus. In support of this idea, it has been shown that partial *nupt* deletions resulting in nuclear activation of a plastid gene can be detected in the laboratory [6]. Furthermore, it has recently been shown that novel nuclear exons can be generated from non-coding organellar DNA sequences [29]. The mechanism of *nupt* deletion described here may therefore be fundamentally important in eukaryotic evolution by providing a significant source of new functional sequences in the nuclear genome.

## Materials and Methods

### Plant Growth Conditions

*Nicotiana tabacum* plants were grown in soil in a controlled environment chamber with a 14 hr light/10 hr dark and 25°C day/18°C night growth regime.

### Analysis of Kanamycin Resistance

Kanamycin selection was performed by plating surface-sterilised seeds on ½ MS salt medium [30] containing 150 µg ml^−1^ kanamycin. Plates were incubated at 25°C with 16 hr light/8 hr dark.

### DNA Extraction

DNA extraction was performed using a DNeasy Plant Mini Kit (Qiagen, Hilden, Germany) according to manufacturer's instructions.

### PCR

Standard PCR was performed in 25 µl reactions with 1.5 U *Taq* DNA Polymerase (New England Biolabs, Ipswich, MA), 1× ThermoPol Reaction Buffer, 10 pmol each primer, 0.5 mM dNTPs, and ∼100 ng genomic DNA or ∼1 ng plasmid DNA template. Cycling was performed with an initial denaturation step at 94°C for 2 min followed by 30 cycles of denaturation at 94°C for 30 sec, annealing at 60°C for 30 sec and extension at 72°C for 1 min. Primers used were neoF and neoR for *neo* PCRs and rbcSF and rbcSR for *rbcS* PCRs. TAIL-PCR was performed as described [31] using the degenerate primer AD1 [32] and specific primers TaadA1, TaadA2 and TaadA3. Standard PCR to extend the ptDNA sequence obtained from TAIL-PCR of kr2.5 was performed using primers TaadA2 and cp130676R as above except that a 3 min extension time was used. See Table 3 for primer sequences.

**Table 3:** Primers used in this study.

Primer name	Sequence (5′–3′)
neoF	TTGAACAAGATGGATTGCACGCAGG
neoR	GAACTCGTCAAGAAGGCGATAGAAGG
rbcSF	GGTGGGCAACTATGCAATGACC
rbcSR	CTTGACGCACGTTGTCGAATCC
aadAF	AGTATCGACTCAACTATCAGAGG
aadAR	GACTACCTTGGTGATCTCGCCTTTC
TaadA1	CCAAGATTTTACCATGAGGGAAGCGGTG
TaadA2	GCCGAAGTATCGACTCAACTATCAGAGG
TaadA3	GTCATCGAGCGCCATCTCGAACCGAC
cp130676R	GGAAGAAATCCGAGTGAATG

### DNA Blot Analysis

For DNA slot blotting, 2 µg of DNA per slot was transferred to Amersham Hybond-N+ membrane (GE Healthcare, Buckinghamshire, UK) using a SRC 072/0 Minifold II slot blotting apparatus (Schleicher & Schuell). Membranes were probed with [^32^P]-dATP labelled probe. Detection and quantification was performed using a Typhoon Trio imaging system and ImageQuant TL software (GE Healthcare, Buckinghamshire, UK). *Neo* and *aadA* probes were generated by PCR using primers neoF and neoR or aadAF and aadAR with pPRV111A::neoSTLS2 [10] as template. pCU5 [33] was used as a ribosomal DNA probe.

### Statistical Analysis

Significance of deviation from an expected Mendelian ratio was determined using a Chi-squared test. Due to the large number of these tests performed, only *P* values <0.01 were considered to be significant in order to minimise the number of false positives.
